# Hepatoprotective Activity of Some Medicinal Plants in Sudan

**DOI:** 10.1155/2019/2196315

**Published:** 2019-12-18

**Authors:** Sumaia A. Ali, Noha H. Sharief, Yahya S. Mohamed

**Affiliations:** ^1^College of Veterinary Medicine, Sudan University of Science and Technology, Department of Veterinary Medicine and Surgery, Khartoum, Sudan; ^2^Faculty of Veterinary Medicine, University of Khartoum, Khartoum, Sudan; ^3^Medicinal and Aromatic Plants Institute and Traditional Medicine, National Centre for Research, Department of Phytochemistry and Taxonomy, Khartoum, Sudan

## Abstract

**Background:**

Liver disorders are common in Sudan and elsewhere. These are traditionally treated by medicinal plants especially in rural areas where they are widely available.

**Methods:**

This review was based on scientific research in hepatoprotective plants performed in Sudan for the period between 2001 and 2016 AD. Data collection was done through scientific evidence of local and international published data, theses, and publications from some libraries in Sudanese universities. Internet was also used to collect published data in different international scientific journals.

**Results:**

In this study, 21 plants from different families were reviewed for the hepatoprotective activity in Sudan. These plants are widely used in traditional medicine for their availability and cheap prices. All of these plants have been scientifically investigated through experimental animal models which confirmed their hepatoprotective activities. This was evaluated by measuring several parameters including liver markers (AST, ALT, ALP, total protein, albumin, and bilirubin) and histopathological investigation. Nineteen (90.5%) of the herbal plants were found to possess significant hepatoprotective activity in animal models. Two (9.5%) of the plants were devoid of this activity. The action of these plants is largely attributed to their phytoconstituents such as flavonoids, antioxidant, and anti-inflammatory effects.

**Conclusion:**

Sudanese herbs may offer novel alternatives to treat liver disorders. Yet determination of the active principle responsible for hepatoprotection needs to be investigated. Further studies on these plants are necessary to establish the efficacy, safety, and exact mechanism of action as a moral alternative in the treatment of liver disorders.

## 1. Background

Sudan possesses a unique variable climatic condition with abundant wealth of flora, cultivated or wild. These are used widely and effectively in folk medicine for treatment of various human and animal aliments, especially by natives in rural areas. In Sudan, medicinal folklore is passed from one generation to another but has not been properly documented [1, 2].

The liver is one of the most important body organs, owing to its multibiological functions in protein, lipid, and carbohydrate metabolism. Acute and chronic liver diseases constitute a global concern, and medical treatments for these diseases are often difficult to achieve and may have limited efficiency [3]. Liver diseases may be fatal posing a serious challenge to international public health [4]. Therefore, there has been considerable interest in complementary and alternative medicines for the treatment of hepatic disorders. Developing therapeutically effective agents from natural products may reduce the risk of toxicity when these drugs are used clinically [5].

The use of herbal medicines to treat liver diseases has increased worldwide, and this is due to the belief that herbal medicines are harmless and free from serious adverse reactions. In addition, they are available and easily obtained from nature. Moreover, the limited therapeutic choices and sometimes unsatisfactory therapeutic failure of modern medicine have increased the usage of alternative medicine including herbal preparations [6, 7].

Worldwide, a great number of remedial plants and their formulations have been claimed to have hepatoprotective activity. Around 160 phytoconstituents from 101 plants have been proved to possess liver-protecting activity [8]. Silymarin, a flavonolignan from “milk thistle” (*Silybum marianum*) plant is used almost exclusively for hepatoprotection [9]. It is known that 80% of the world population are using medicinal plants and 30% are prescribed by physicians [10]. In Sudan, it is estimated that over 90% of the population depend on plant-derived medicines [11].

Plants derived natural products such as flavonoids, terpenoids and sterols have received considerable attention in recent years due to their diverse pharmacological properties including antioxidant and hepatoprotective activity [12].

*In vivo* and *in vitro* evaluation models have been developed for the ability of the plants to prevent or cure liver toxicity in laboratory animals induced by various hepatotoxins [13]. The prevention of liver damage induced by CCl_4_ has widely been used as a marker of hepatoprotective activity of drugs in general [14].

Regardless of the fact that quite a number of Sudanese plant species are internationally renowned for high quality and commercial viability, they have not been fully exploited, probably due to lack of effective marketing system [2]. In the Folk medicine of Sudan, numerous plants were used effectively in the treatment of jaundice and different liver disorders, but in most cases their efficacy has not received any comprehensive scientific evaluation. In the present review, scientific literature evaluating the hepatoprotective activity of some Sudanese medicinal plants using different hepatotoxicity models has been discussed.

## 2. Methods

This review aimed at compiling data on hepatoprotective plants in Sudan published locally and internationally during the period between 2001 (starting point of scientific research about hepatoprotective herbs) and 2016 AD. In this review, the hepatoprotective herbs were listed according to scientific information obtained on their medicinal use for curing liver disorders, reported in publications and theses from some libraries in Sudanese universities. Internet was also used to collect published data in different international scientific journals through PubMed and Google Scholar search engines. Local traditional medicinal books such as [1, 2, 15, 16] were used to collect information about distribution and traditional uses of these plants. Literature search described the use of well-established experimental models (*in-vivo* and *in-vitro*) such as carbon tetrachloride (CCl_4_)-induced liver injury [17, 18] and paracetamol-induced liver injury [19] using experimental animals or cell line. Comparison is made between the efficacy of the herbal drug and that of conventional drugs used to treat liver disease particularly silymarin [9, 20]. The liver enzymes aspartate transaminase (AST), alanine transaminase (ALT), alkaline phosphatase (APT), total bilirubin, total protein (TP), and albumin (Alb) were used as biomarkers to estimate the level of hepatocyte damage. Review also included the histopathological evaluation of the effect of the standard hepatotoxic agents compared with that of the plant extracts used as hepatoprotective agents.

Briefly, this review summarizes the scientific information of 21 hepatoprotective herbal drugs used in Sudan traditional medicine for the treatment of liver disorders including their botanical name, family, part of the plant used, local name, distribution, chemical constituents, traditional uses, the extracts used, the dosage of extracts, the model used, parameters estimated, histopathology, and the results of hepatoprotective studies on each plant. The possible mechanism of action of these hepatoprotective plants has been also discussed.

## 3. Results

Twenty-one Sudanese medicinal plants from different families were reviewed for the hepatoprotective activity in Sudan. These plants are widely used in traditional medicine especially in rural areas for their effectiveness, availability, and cheap prices.

These plants have been scientifically evaluated using different experimental models especially CCl_4_ and paracetamol to induce liver damage. These plants were assessed by measuring several parameters including liver markers (AST, ALT, ALP, total protein, albumin, and bilirubin) and histopathological investigation. Nineteen (90.5%) of the medicinal plants were found to possess significant hepatoprotective activity in animal and cell line models. Two (9.5%) of the plants were devoid of this activity.

**Figure 1:**
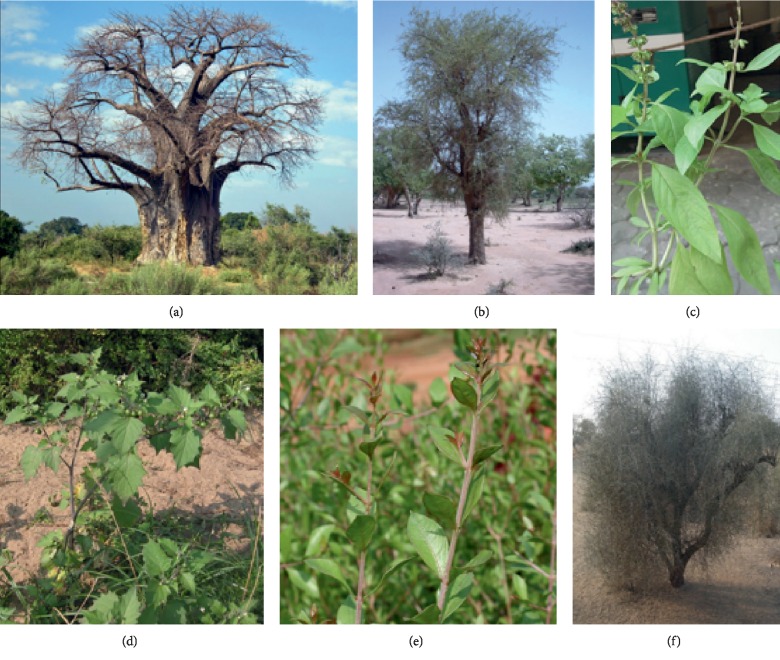
Some Sudanese medicinal plants (source: Wikimedia Commons): (a) *Adansonia digitata*, (b) *Balanites aegyptiaca*, (c) *Occimum basilicum*, (d) *Solanum nigrum*, (e) *Lawsonia inermis*, and (f) *Capparis decidua*.

Traditional plants commonly used to treat liver disorders in Sudan, their distribution, chemical composition, part of the plant used, method of preparation, and outcome of treatment are shown in Tables 1 and 2 and Figure 1.

### 3.1. *Acacia mellifera* (Vahl.), Fabaceae, Keka/Kitir

The hepatoprotective activity of *A*. *mellifera* leaves ethanolic extract and fractions were investigated against DCFH- (dichlorofluorescein) and CCl_4_-induced hepatotoxicity on cultured liver cells and rats [21]. DCFH-toxicated cells were recovered to about 100% with 100 *μ*g/ml of *A*. *mellifera* (AM) crude extract, and supplementation with 200 *μ*g/ml of this further enhanced the hepatocyte proliferation by about 20%. Ethyl acetate, aqueous, and n-butanol fractions showed the most promising effective hepatoprotection, while hexane and dichloromethane fractions were devoid of this activity.

In CCl_4_ rat model, administration of AM ethanolic extract at a dose of 250 and 500 mg/kg.b. wt orally and CCl_4_ in liquid paraffin (1 : 1, 1.25 ml/kg) intraperitoneally (IP) for three weeks significantly normalized alkaline phosphatase (ALP), bilirubin (Bil), cholesterol, triglyceride, and lipoprotein levels and elevated tissue nonprotein sulphhydryl and total protein. The histopathology showed that AM at a dose of 250 mg/kg revealed congested hepatic central veins with mild hepatocyte necrosis and fatty changes. Rats receiving 500 mg/kg of AM or silymarin showed normal hepatocytes and central veins.

The phytochemical screening of the fractions showed the presence of alkaloids, flavonoids, polyphenolic tannins, sterols, and saponins. The antioxidant activity of AM fractions was also performed against 1,1-diphenyl-2-picrylhydrazyl (DPPH) radical. The highest antioxidant activity was found in ethyl acetate and n-butanol followed by dichloromethane, hexane, and aqueous extracts.

The hepatoprotective activity of the plant may be attributed to the phenolic compounds, flavonoids, and saponins [61–64]. Hepatoprotective activity of flavonoids is due to their ability to scavenge free radicals [62].

### 3.2. *Adansonia digitata* L., Malvaceae, Baobab Tree

The hepatoprotective effect of the methanolic extract of *Adansonia digitata* L. fruit pulp (100 and 200 mg/kg) was investigated against CCl_4_-induced hepatotoxicity in rats. Silymarin (25 mg/kg) was used as a reference drug. The two doses of *A. digitata* showed dose-dependant hepatoprotective effects on CCl_4_-induced hepatotoxicity in rats. This was clearly seen by a significant decrease (*P* < 0.05) in the serum of AST, ALT, ALP, and bilirubin as well as less pathological changes in liver sections compared with the CCl_4_-treated group.

The protection of *A. digitata* L. fruit pulp against CCl_4_-induced liver damage and restoration of biochemical values could result from the fruit content of triterpenoids [65], *β*-sitosterol, *β*-amyrin palmitate or/and *α*-amyrin, and ursolic acid along with the antioxidant, anti-inflammatory, analgesic, immunostimulant, and antimicrobial activities of *A. digitata* L. fruit pulp [66].

### 3.3. *Argemone mexicana* L., Papaveraceae, Khash Khash

The hepatoprotective activity of water and methanolic extracts of *Argemone mexicana* L. aerial part was evaluated against CCl_4_ damage in rats. The hepatic injury was achieved by injection of CCl_4_ 3 ml/kg s.c in olive oil (1 : 1 v/v). The extracts were given at different doses, 100, 200, and 400 mg/kg/day for methanolic extract and 400 mg/kg/day orally for aqueous extract for 5 days. CCl_4_ was injected at the 3^rd^ day. The methanol extract at 100 mg/kg offered significant hepatoprotective activity (*P* < 0.05) by reducing serum ALT, AST, and ALP.

The results of the histopathology of methanol extract at a dose of 100 mg/kg showed healing of the liver parenchyma and regeneration of liver cells. The results were comparable with silymarin (70 mg/kg orally). The mechanism by which *A. mexicana* displays hepatoprotection is not known yet [24].

### 3.4. *Anogeissus leiocarpus* (*DC) Wall.*, Combretaceae, Sahib

Hepatoprotective effects of the *Anogeissus leiocarpus* bark ethanolic extract were investigated in rats against carbon tetrachloride- (CCl_4_-) induced liver injury [40]. Rats received the plant extract three times at 0, 12, and 24 hours. CCl_4_ was injected 1.25 ml/kg as a single dose 30 minutes before the first dose of test extracts.

Blood samples were collected after 36 h for haematological and biochemical investigation before the rats were euthanized, and liver samples were taken for histopathology.

Phytochemical screening of the *A. leiocarpus* bark ethanolic extract indicated the presence of tannins, saponins, flavonoids, sterols, triterpenoids, and cumarins. Oral administration of the ethanolic extract of the plant at a dose of 200 mg/kg displayed a significant (*P* < 0.05) hepatorenal protective effect against CCl_4_ by lowering liver biomarkers (AST, ALT, and ALP), kidney biomarker levels (urea and creatinine), as well as normalizing haematological parameters when compared with standard drug silymarin. Histopathological investigation of liver and kidney tissues verified the protective effect of the plant extract.

The protective effect of the extract may be related mainly to the antioxidant property of its high content of flavonoids, tannins, sterols, and triterpenes [67–69].

### 3.5. *Balanites aegyptiaca* L., Balanitaceae, Hegleig Tree (Ar) Laloub Fruit

The hepatoprotective effects of water and methanolic extracts of *Balanites aegyptiaca* bark were investigated in rats injected with 0.2 ml/kg carbon tetrachloride (CCl_4_) for 10 days. The extracts were administered at the same time with CCl_4_ at a dose of 250 to 500 mg/kg orally for 10 days. Significant decreases in AST, ALT, and ALP activities, bilirubin concentration, as well as mild hepatocyte lesions were noticed in rats administered with plant extracts compared with the CCl_4_ group [25].

Phytochemical screening of aqueous stem bark extracts of *B. aegyptiaca* indicated the presence of alkaloids, flavonoids, glycosides, phenols, saponins, and tannins [70]. Flavonoids, phenolic compounds, and saponins are known to have antioxidant and hepatoprotective effects. Furthermore, the flavonoids and saponins of *B. aegyptiaca* may stabilize reactive oxygen species by reacting with them and oxidizes subsequently to more stable and less reactive radicals [70, 71].

### 3.6. *Cannabis sativa* L., Cannabaceae, Bango, Hashish

The hepatoprotective activity of *Cannabis sativa* L. against CCl_4_-induced hepatotoxicity in rats was investigated [26]. Daily oral doses of *C. sativa* L. oil 1 and 0.5 ml/kg body weight were administered to rats. The hepatotoxicity induced by injecting CCl_4_ in paraffin oil (1 : 9 v/v) at a dose of 0.2 ml/kg for 10 days was found to be inhibited by simultaneous oral administration of *C. sativa* oil of at a dose of 1 and 0.5 ml/kg. This was evidenced by the decreased levels of serum AST, ALT, ALP, and bilirubin. However, the liver changes were also inhibited compared with control rats and silymarin group. The oil of *C. sativa* contains a hepatoprotective ingredient that protects the liver from carbon tetrachloride damage [26].

### 3.7. *Capparis decidua* (Forsk.), Capparaceae, Tundub

The aqueous and methanolic extracts of *Capparis decidua* stems locally known as Altoundob were screened for their hepatoprotective activity against CCl_4_-induced hepatotoxicity in rats. The hepatotoxicity produced by administration of CC1_4_ in paraffin oil (1 : 9 v/v) at a dose of 0.2 ml·kg^−1^ for 10 days was found to be inhibited by simultaneous oral administration of aqueous and methanolic extracts of C. *decidua* stems (200, 400 mg·kg^−1^ b.wt.) for 10 days, with evidence of decreased level of serum AST, ALT, ALP, and bilirubin. In addition, the concurrent administration of both extracts with CC1_4_ for 10 days masked the liver fatty changes induced by the hepatotoxic compound observed in the intoxicated control rats. The results were comparable with the hepatoprotective effect of the standard drug silymarin. The results showed that the aqueous extract of *C. decidua* had higher activity than the methanolic extract, probably related to more polar phytoconstituents [7, 27].

The preliminary phytochemical screening of the powdered plant showed the presence of alkaloids, flavonoids, tannins, sterols, saponins, cyanogenic glycosides, and cumarins as major constituents of the studied extracts.

The study revealed the evidence of the presence of flavonoids, cyanogenic glycosides and triterpenes which are antioxidants and may be responsible for the hepatoprotective property [13, 72–74].

In addition, *C. decidua* was found to alter superoxide dismutase and catalase enzyme levels to reduce oxidative stress. Moreover, the high contents, in the plant, of minerals such as iron and vitamins such as vitamin C may contribute to the extract hepatoprotectivity [75].

### 3.8. *Combretum hartmannianum* (Schweinf), Combretaceae, Habiel. Habeel Al Gabal

The hepatoprotective effect of *Combretum hartmannianum* leaves methanolic extract was investigated against CC1_4_- and paracetamol-induced hepatotoxicity. The methanolic extract of *C. hartmannianum* (12.5, 25, 50 mg/kg) was injected intraperitoneally (IP) one hour before injection of CC1_4_ (800 mg/kg IP) and paracetamol (1 g/kg P.O). The results were compared with silymarin, a standard hepatoprotective drug. *C. hartmannianum* leaves' extract showed hepatoprotective effect against CC1_4_ induced hepatotoxicity which was evidenced by significant decrease in AST, ALT, ALP, and bilirubin and significant increase in serum total protein and albumin. Significant decreases in AST and ALT were also observed in rats given *C. hartmannianum* leveasthe methanolic extract of *C. hartmannianum* leaves IP one hour before oral administration of paracetamol to induce hepatotoxicity [28]. The methanolic extract of *C. hartmannianum* leaves contained flavonoids, tannins, saponins and unsaturated sterols and no cumarins, alkaloids, and triterpenoids [28]. The hepatoprotective effect of this plant may be due to high antioxidant activity in DPPH free radical scavenging assay and flavonoid content [76].

### 3.9. *Dobera glabra* (Forsk.), Salvadoraceae, Al Meikah

*Dobera glabra* leaves' aqueous and methanolic extracts were investigated for their hepatoprotective activity against CCl_4_-induced hepatotoxicity in rats. CC1_4_ was injected in paraffin oil (1 : 9 v/v) at a dose of 0.2 ml·kg^−1^ for 10 days. Leaf extracts were given by simultaneous oral administration at a dose of 200 and 400 mg·kg^−1^ b.w. for 10 days. ALT, AST, and bilirubin were significantly elevated in plant extract groups compared with standard drug silymarin and CC1_4_ group. Moreover, administration of both extracts with CC1_4_ for 10 days produced diffuse centrilobuar hepatocyte necrotic lesions in hepatocytes [30].

The preliminary phytochernical screening of the powdered plant showed the presence of alkaloids, flavonoids, tannins, sterols, saponins, cyanogenic glycosides, and cumarins as major constituents of the studied extracts [30].

### 3.10. *Khaya senegalensis* (Desr.), Meliaceae, Mahogany Tree

The hepatoprotective activity of the aqueous extract of the bark of *Khaya senegalensis* was evaluated against CCl_4_-induced liver damage in rats. Bark extract was administered at a dose of 250 and 500 mg/kg for 5 days, while CCl_4_ (3 ml/kg body weight/rat) was injected subcutaneously on the 3^rd^ day of experiment. Silymarin (50 mg/kg) was also used as a standard drug. The levels of AST, ALT, ALP, and bilirubin in groups treated by the extract were significantly increased compared with that of the silymarin group; the hepatoprotective activity was supported by histopathological finding of liver tissue [30].

The hepatoprotective effect of the methanolic extract of the bark of Sudanese plant *Khaya senegalensis*, which is used in folk medicine for treatment of jaundice, was investigated. The hepatoprotective effect was tested in rats against CCl_4_ and paracetamol-induced hepatotoxicity. The extracts were given intraperitoneally one hour before injection of CCl_4_ (800 mg/kg IP) and administration of paracetamol (1 and 2 g/kg P.O). The results were compared with the standard hepatoprotective agent silymarin. The methanolic extract of the bark of *K. senegalensis* showed a hepatoprotective effects against CCl_4_ and paracetamol-induced hepatotoxicity, as evidenced by the significant decrease in ALT, AST and ALP. Moreover, dichloromethane and petroleum ether extracts against paracetamol-induced hepatotoxicity did not possess hepatoprotective effect as evidenced by failure to decrease ALT and AST activities. Chloroform and ethyl acetate extracts showed significant hepatoprotective effect against toxicity induced by paracetamol as evidenced by significant decrease of ALT and AST. The mild histopathological lesions observed in the groups treated with methanolic and chloroform extracts of the bark of *K. senegalensis* compared with paracetamol groups indicated some protective effect [32, 33].

*Khaya senegalensis* bark aqueous extract was also evaluated for its hepatoprotective activity against CCl_4_-induced liver damage in rats [31]. The bark of *K. senegalensis* was orally administered at a dose of 250 and 500 mg/kg for five days. The levels of AST, ALT, ALP, bilirubin, total protein, and albumin, in groups treated by the extract were significantly decreased compared with that of CCl_4_ group. These findings were strongly supported by the histopathological results of liver sections in comparison with the group treated by the standard drug silymarin. This study indicates that, the aqueous extract of *K. senegalensis* bark possesses hepatoprotective ingredients [31].

The hepatoprotective activity of *K. senegalensis* bark ethanolic extract was evaluated against CCl_4_ in rats [23]. The extract was given three times (0, 12, and 24 hours) at a dose of 200 mg/kg orally. CCl_4_ (1.25 ml/kg) diluted in liquid paraffin (1 : 1) was injected subcutaneously 30 minutes after the administration of the first dose of vehicle as single dose. After 36 h, blood samples were collected for biochemical and haematological investigations. The extract showed significant decrease in the level of serum enzymes ALT, AST, and ALP, as well as significant decrease in the concentration of total and direct bilirubin compared with the CCl_4_ group. The extract also masked CCl_4_-induced enlargement of the liver which confirmed the hepatoprotective effect of the plant extract.

The preliminary phytochemical screening of the powdered material of plants revealed the presence of tannins, sterols, saponins, cumarins, and triterpenes. The plant material was devoid of alkaloids, flavonoids, and anthraquinone compounds. The extract exerts weak antioxidant activity when tested by DPPH assay [23].

### 3.11. *Kigelia africana* (Lam.), Bignoniaceae, Umm Shutour

The hepatoprotective activity of aqueous and methanolic extracts of *Kigelia africana* was investigated against CCl_4_-induced liver damage on male Wistar rats. CCl_4_ was injected at a dose of 3 ml/kg, subcutaneously (1 : 1 dilution with olive oil), on the 3^rd^ day of experiment. Silymarin was given orally (50 mg/kg/day) for 5 days. Methanolic extract was given at a dose of 100, 200, and 400 mg/kg/day, while the aqueous extract was given at a dose of 400 mg/kg/day for 5 days. The study revealed that, administration of the two extracts (aqueous and methanol) of the plant seeds has toxic effects which resulted in alterations in haematological parameters (Hb, WBCs MCH, MCHC, and granulocytes) and also alterations in AST, ALT, and ALP activities. There were also histopathological alterations in the liver and kidney [34].

### 3.12. *Lawsonia inermis* L., Lythraceae, Henna

The hepatoprotective effect of *Lawsonia inermis* leaves' methanolic extract on CCl_4_-induced hepatotoxicity in rats was investigated. The leaves of *L. inermis* methanolic extract, which are obtained by maceration, were administered orally at a dose of 100 mg/kg and 200 mg/kg. Silymarin (25 mg/kg), a potent hepatoprotective drug, was used as the standard control. The two doses of the plant extract showed dose-dependent hepatoprotective effects, as evident by the significant reduction (*P* < 0.05) in serum levels of AST, ALT, ALP, and bilirubin along with the improvement in histopathological liver sections compared with CCl_4_-only treated animals. The results indicated that this plant material could provide a hepatoprotective effect that could be attributed to its antioxidant properties [35]. It has been reported that the plant extract affords hepatoprotective activity due to its antioxidant property attributed to the flavonoid contents that are effective scavengers of superoxide anions, peroxynitrite, peroxyl, and hydroxyl radicals [77].

### 3.13. *Lepidium sativum* L., Brassicaceae, Pepper Cress or ELRshad

The protective effect of *Lepidium sativum* L. seeds' methanolic extract (200 and 400 mg/kg) was investigated against CCl_4_-induced liver damage in rats. CCl_4_ was injected simultaneously with the extracts at a dose of 0.2 ml/kg after dissolving in paraffin oil (1 : 9 v/v) for 10 days. Mean serum AST, ALT, ALP, and bilirubin concentration was significantly reduced in rats given *L. sativum* extract. However, the severe fatty changes that shown in the liver sections of rats given CCl_4_ were significantly decreased in plant-treated groups. The mechanism of the hepatoprotective action of the plant is uncertain but may be due to inhibition of lipid peroxidation in the liver.

The presence of flavonoids, triterpens, alkaloids, tannins, and coumarins in *L. sativum* explains its role in hepatoprotection by inhibiting the free radical-mediated damage. In addition, flavonoids, triterpenes, and tannins were antioxidant agents and interfere with free radical formation [36].

### 3.14. *Moringa oleifera* Lam., Moringiaceae, Al-rawag

Hepatoprotective activity of *Moringa oleifera* leaves aqueous extract was investigated against CCl_4_-induced liver injury in rats. Rats received CCl_4_ in paraffin oil (1 : 9 v/v) at a dose of 0.2 ml·kg^−1^ for 10 days to induce hepatocellular damage. *M. oleifera* extract was given concurrently with CCl_4_ at a dose of 200 and 400 mg·kg^−1^; p.o. The simultaneous administration of the aqueous extract with CCl_4_ for 10 days reduced the levels of serum AST, ALT, ALP, and bilirubin. Moreover, the liver fatty change observed in the intoxicated control rats was also reduced especially at a dose of 200 mg·kg^−1^. Degenerative changes with cytoplasmic rarefication and acidophilic cytoplasm with pycknotic nuclei were observed precentrally in the livers of rats that received *M. oleifera* leaves' aqueous extract at a dose rate of 400 mg·kg^−1^. The results of this study indicated that the aqueous extract of *M. oleifera* leaves could afford significant protection against CCl_4_-induced liver injury in rats especially at lower dose [37]. Furthermore, the preliminary phytochemical study revealed the presence of alkaloids, saponins, flavonoids, tannins, sterols, glycosides, and cumarins [37]. It has been reported that a number of phytoconstituents have protective effects on liver due to antioxidant properties such as flavonoids, triterpenoids, and sterols. Presence of those compounds in *M. oleifera* may be responsible for the protective effect on CCl_4_-induced liver damage in rats [12, 78]. Also, a number of scientific studies reported that *M. oleifera* is known to be a source of antioxidants due to its total phenolic, vitamin A, and vitamin E contents which is known to reduce lipid peroxidation [79, 80].

### 3.15. *Nigella sativa* L., Ranunculaceae, Black Seed, Black Cumin, or Habat Elbarka

The protective effects of *Nigella sativa* seed extract (NSSE) against acetaminophen- (APAP-) induced hepatotoxicity in TIB-73 cells and rats were investigated. Toxicity in TIB-73 cells was induced with 10 mmol/L APAP, and the protective effects of NSSE were evaluated at 25, 50, 75, and 100 mg/mL. For *in vivo* examination, a total of 30 rats were equally divided into five experimental groups: normal control (vehicle), APAP (800 mg/kg body weight single IP injection) as a hepatotoxic control, and three APAP and NS pretreated (2 weeks) groups (APAP + NSSE 100 mg; APAP + NSSE 300 mg; and APAP + NSSE 900 mg/kg). TIB-73 cell viability was drastically decreased by (49.0 ± 1.9)% after 10 mmol/LAPAP treatment, which also increased reactive oxygen species production. Cotreatment with NSSE at 25, 50, 75, and 100 mg/mL significantly improved cell viability and suppressed reactive oxygen species generation. *In vivo*, the APAP induced alterations in blood lactate levels, pH, anionic gap, and ion levels (HCO3−,Mg2+, and K+), which tended to normalize with the NSSE pretreatment. The NSSE also significantly decreased serum ALT, AST, and ALP, which correlated with decreased levels of hepatic lipid peroxidation (malondialdehyde), increased superoxide dismutase levels, and reduced glutathione concentrations. Improved hepatic histology was also found in the treatment groups other than the APAP group [39]. The protective effects of *N*. sativa seed extract against APAP-induced hepatotoxicity and metabolic disturbances in rats might be related to improved antioxidant activity and attenuation of oxidative stress, lipid peroxidation, and ROS generation.

Aqueous extract of *N. sativa* seeds was tested for its hepatoprotective effect against CCl_4_-induced hepatotoxicity in rats. Plant extract was given at a dose of 250 and 500 mg/kg for 5 days. CCl_4_ was injected (3 ml/kg) subcutaneously on the 3^rd^ day. Silymarin (50 mg/kg) was used as a reference hepatoprotective drug. Rats were scarified after 5 days. The levels of serum AST, ALT, ALP, and total protein (TP) were significantly decreased in groups treated with extract (250 and 500 mg/kg) compared with that of CCl_4_. The biochemical observations were correlated with histopathological examination of rat liver sections [30].

In another study, *N. sativa* methanolic seeds' extract was investigated in CCl_4_-induced liver damage in rats. Rats were IP administered with CCl_4_ (0.2 ml·kg^−1^ dissolved in liquid paraffin 1 : 9) together with 250 and 500 mg·kg^−1^ of the methanolic extract of *N. sativa* which was dissolved in dimethylsulfoxide. Rats were scarified after 10 days. The levels of ALT, AST, and ALP were found to be significantly higher in both CCl_4_- and *N. sativa*-treated groups compared with the controls, but the increase was less in the groups which were treated with *N. sativa* methanolic extract. The histopathological changes in the livers of the CCl_4_ group exhibited severe centrilobular vacuolation and congestion but in the groups treated with 250 and 500 mg·kg^−1^ b. wt., these changes were to a lesser extent [38].

### 3.16. *Ocimum basilicum* L., Lamiaceae, Alryhan

The hepatoprotective activity of *Ocimum basilicum* L. whole plant ethanolic extract was evaluated. The extract was tested on rats at an oral dose of 200 mg/kg for hepatoprotective effect before injection of CCl_4_ (0 h) and following posttreatment with CCl_4_ at 12 and 24 hours. Rats received the *O. basilicum* extract three times at 0, 12, and 24 hours, while the CCl_4_ was injected once at a dose of 1.25 ml/kg 30 minutes before the first dose of test extracts.

Blood samples were collected after 36 h for haematological and biochemical investigation before the rats were euthanized, and liver samples were taken for histopathology.

*O. basilicum* ethanol extract at a dose of 200 mg/kg exhibited a significant (*P* < 0.05) protective effect by lowering serum levels of AST, ALT, and ALP comparable with that of silymarin used as a standard drug compared with CCl_4_. Haematological parameters were also found similar to the silymarin group. These biochemical observations were supplemented by histopathological examination of liver which proved to be protected by the plant extract. The study concluded that *O. basilicum* ethanolic extract has a potential hepatoprotective activity against CCl_4_-induced hepatotoxicity in rats [40].

Phytoconstituents identified in *O. basilicum* whole plant ethanolic extract included flavonoids, alkaloids, tannins, saponins, triterpenes, sterols, and cumarins.

The protective effect of the extract probably related to the antioxidant property due to its high content of flavonoids, saponin, tannins, sterols, and triterpenes and due to its superoxide radical and nitric oxide radical scavenging activities [81–83].

### 3.17. *Phoenix dactylifera* Linn*.,* Palmae Date, Date Palm

The hepatoprotective effect of Sudanese dates palm (*Phoenix dactylifera* Linn) pollen grain powder (produced from Elgolid Locality, Northern State of Sudan) in albino rats was evaluated. The hepatotoxicity was achieved by injecting CCl_4_ subcutaneously (3 ml/kg dissolved in equal preparation with olive oil 1 : 1) on the 3^rd^ day. Date palm pollen grains were given orally at a dose of 250 and 500 mg/kg for five days. Silymarin was given orally (50 mg/kg) as a reference drug for 5 days. Date palm pollen grain powder showed significant (*P* < 0.05) hepatoprotective activity which was indicated by decreased of ALT, AST, and ALP levels of treated groups compared with CCl_4_. These results correlated with histopathological changes which proved centrilobular necrosis with slight congestion [41].

The mechanism by which the date palm fruit extract induced its hepatoprotective activity is not certain. However, it could be due to *β*-sitosterol, a constituent of *P. dactylifera* L. which may be partly responsible for the protective activity against hepatotoxicity [84]. It is suggested that flavonoids in *P. dactylifera* L. could be contributing as a factor to its ability for hepatoprotection through inhibition of cytochrome P-450 aromatase [85]. In addition, the high content of vitamin C in the date palm pollen grains may also play a role in hepatoprotection [41].

### 3.18. *Raphanus sativus* L., Cruciferae, Alfgel

*Raphanus sativus* L. water and methanolic extracts were investigated for a possible hepatoprotective activity against carbon tetrachloride-induced hepatotoxicity in rats. CCl_4_ (0.2 ml/kg in paraffin oil 1 : 9 v/v) was administered IP with either methanolic or water extract at doses of 200 and 400 mg·kg^−1^. The animals were sacrificed after 10 days. Biochemical results showed that CC1_4_ induced hepatotoxicity which was reduced by the use of the plant as indicated by inhibition of the increased serum AST, ALT, and ALP activities and bilirubin concentration besides histopathological changes. The phytochemical tests revealed presence of triterpenes, alkaloids, flavanoids, tannins, saponin, and cournarins but negative for cyanogenic glycosides and anthraquinone glycosides [42].

### 3.19. *Solanum nigrum* L., Solanaceae, Enab Eldib

The hepatoprotective effects of *Solanum nigrum* water and methanolic extracts were investigated in rats injected with 0.2 ml/kg CCl_4_ for 10 consecutive days. *S. nigrum* extracts were administered orally at a dose of 250 to 500 mg/kg for 10 days. The extracts showed a hepatoprotective effect against CCl_4_-induced liver damage, which was evident by the decrease in serum AST, ALT, and ALP activities and bilirubin concentration and by mild histopathological lesions when compared with the group of rats injected with CCl_4_ alone. The water extract appears to have a better hepatoprotective effect than the methanolic one which could be due to more polar phytoconstituents [43].

### 3.20. *Sterculia setigera* Del., Sterculiaceae, Terter

The ethanolic and ethyl acetate extracts of *Sterculia Setigera* stem bark that belongs to the family of Sterculiaceae was evaluated for its hepatoprotective activity in albino rats with liver damage induced by carbon tetrachloride (CCl_4_). The hepatotoxicity was produced by the administration of CCl_4_ at a dose of 0.2 ml/kg for 10 days. The extracts of *S. Setigera* stem bark were given orally at a dose of 200 and 400 mg/kg body weight. The extracts exhibited moderate protective effect by lowering the serum levels of ALT, AST, ALP, total protein, albumin, and bilirubin concentration to significant extent. In addition, the concurrent administration of the plant extracts with CCl_4_ for 10 days masked the liver changes induced by the hepatotoxic compound in rats and compared with hepatoprotective effect of the standard drug Silymarin. However, severe necrotic hepatic lesions induced by CCl_4_ were reduced by ethanolic and ethyl acetate extract of the plant [44].

### 3.21. *Tamarindus indica* L., Caesalpinaceae, Aradaib

The fruit pulp ethanolic extract of *Tamarindus indica* was investigating in protecting liver damage induced by CCl_4_. Ethanolic extract of *T. indica* was administered at a dose of 150 mg/kg/day for 5 days. Silymarin (50 mg/kg) was used as a standard drug. CCl_4_ was injected subcutaneously at 0.2 ml/kg diluted (1 : 9) in liquid paraffin on day 2 and 3 and received saline orally for 5 days. Results indicated that, the ethanolic extract of *T. indica* ameliorated the damage caused by CCl_4_ by lowering the levels of AST, ALT, and ALP and the concentration of bilirubin, and this effect was verified by improvement of histopathological picture in the livers of the group treated by the plant compared with the liver of the CCl_4_ group which exhibited severe centrilobular necrosis and fatty vacuolation. The study indicates that *T. indica* ethanolic extract possess hepatoprotective ingredients [45].

The presence of moderated concentration of tannins and alkaloids and low concentration of flavonoids and saponin in the ethanolic extract of the fruit pulp of *T. indica* could be responsible for the membrane-stabilizing activity [86].

## 4. Discussion

In Sudan, medicinal plants possess a distinctive position in folk medicine as well as their important position in the sociocultural and spiritual arena of rural and tribal life in Sudan. Throughout Sudan's long history, local indigenous cultures have merged with many other cultures such as Pharaonic, Christian, and Islamic. This has helped traditional medicine and the use of medicinal plants to become an important part of Sudan's cultural heritage [87].

In Sudan, people in rural areas prefer treatment of various diseases by medicinal plants than by modern synthetic drugs which are expensive and because of difficulty to access medical services particularly in areas of conflicts and political instability [88]. This review presents data on 21 Sudanese herbs evaluated scientifically as hepatoprotective using well-known experimental models. From this review, it is clear that the medicinal plants play an important role in the treatment of a variety conditions including liver diseases. Most medicinal plants are widely distributed in the rural areas. Carbon tetrachloride and paracetamol as hepatotoxins are used widely to assess hepatoprotective plants in Sudan.

The hepatoprotective activity of these plants is based on lowering liver biomarkers as well as suppressing of degenerative changes in hepatocytes produced by the hepatotoxic agent compared with reference hepatoprotective drug (silymarin). The evaluation is usually based on administration of different doses of plant extracts using aqueous or chemical extracts such as methanol, ethanol, petroleum ether, and ethyl acetate.

The majority of reports used either methanol or aqueous extracts (alone or with other solvents), with the exception of eight studies in which both extracts were evaluated by similar protocols and dosages (*C. decidua, B. aegyptiaca, A. Mexicana, S. nigrum, M. oleifera, R. sativus, D. glabra,* and *K. africana)*. In positive plants (*C. decidua, B. aegyptiaca, A. Mexicana, S. nigrum, M. oleifera,* and *R. sativus*), the studies investigated the aqueous and methanolic extracts of *C. decidua, S. nigrum,* and *M. oleifera* and clearly concluded that the aqueous extracts have better activity compared with methanolic one especially in lower doses [7, 27, 37, 43]. In contract, the study by [24] indicated that the hepatoprotective effect of *A. mexicana* is more pronounced in low dose (100 mg/kg) of methanolic extract than the aqueous extract. But in much higher dosage (400 mg/kg), the aqueous extract was more effective.

The activity of *R. sativus* and *B. aegyptiaca* in both extracts seem to be comparable and the higher dosages are the best [25, 42]. Two studies evaluated hepatoprotective activity of aqueous and methanolic extracts of the leaves of *Dobera glabra* and the seeds of *Kigelia Africana*. Both plant extracts were not active at the dosage used, and rather seemed to have hepatotoxic effects [29, 34]. The biochemical parameters used to verify the activity of both plant extracts did not differ statistically from the CCl_4_ group, and the severe necrotic hepatic lesions induced by CCl_4_ were not reduced to significant level by the administration of the extracts.

The mechanisms of action as hepatoprotective might be due to antioxidant properties of these plants which have major role in protecting liver cells from oxidative stress. Moreover, available information on this review clearly showed that these herbs are richest of phytoconstituents such as coumarins, terpenoids, glycosides, flavonoids, alkaloids, saponins, and tannins which are well known as hepatoprotective agents [27].

One of the noteworthy weaknesses in the design of most of the cited studies is that the methods used to test the hepatoprotective properties of these plants that most of the studies used cotreatment and pretreatment regimens (meaning that the plant extracts were given either before, or with, CCl_4_ and paracetamol). This is problematic for two reasons: First, both CCl_4_ and paracetamol toxicity depend upon conversion to reactive metabolites by cytochromes P450. Obviously, pretreating or cotreating with a milieu of phytochemicals has great potential to interfere in that metabolism [19]. Thus, such experiments may not actually demonstrate that the plants are hepatoprotective, but simply that they compete for drug-metabolizing enzymes. Second, pretreatments are not clinically relevant. A disease cannot be treated before it develops.

Further investigation should be done to evaluate the posttreatment efficacy of these plants using animal experimental models against CCl_4_- and paracetamol-induced liver damage. Future studies also should elucidate the exact mechanisms of actions of the active plants. Clinical trials have to be conducted to evaluate efficacy, safety, proper dosage, and pharmacokinetic aspects of the active plant extracts in order to develop safe and effective dosage forms from these plants.

## 5. Conclusion

This review shows that some Sudanese medicinal plants have a crucial role in protecting liver from chemical injuries using *in vivo* and *in vitro* models. Herbal drugs in Sudan play a vital role in the primary health care since 90% of Sudanese people use medicinal plants to treat different diseases including liver disorders [11], due to high cost and inadequacy of conventional drugs used in the treatment of liver diseases. The action of these plants may be due to phytoconstituents, antioxidant, and anti-inflammatory effects. Further studies on these plants are necessary to establish the efficacy, safety, and exact mechanism of action as a moral alternative in the treatment of liver disorders.

Advanced tools and equipment are needed for identification, isolation, and purification of the active ingredients of the hepatoprotective plants and to examine their efficacy and safety through controlled clinical trials. There should be a motivation plan to Medicinal Plants Research Centre in Sudan to create cooperative collaborative research activities among the involved institutions.

## Figures and Tables

**Table 1:** Sudanese hepatoprotective herbs.

Botanical names	Part used	Extracts used/dosage	Model(s) used	Parameters estimated	Histopathology	References
1. *Acacia mellifera* (Vahl)	Leaves	Ethanol (250 and 500 mg/kg)	CCl_4_-induced	AST, ALT, GGT, ALP, bilirubin, TP	Cured the tissue lesions	[21]

2. *Adansonia digitata* L.	Fruit's pulp	Methanol (100, 200 mg/kg)	CCl_4_-induced	AST, ALT, ALP, bilirubin	Minimal necrosis and regeneration of hepatocytes compared with CCl_4_	[22]

3. *Anogeissus leiocarpus (*DC) Wall	Bark	Ethanol (200 mg/kg)	CCl_4_-induced	AST, ALT, ALP, bilirubin, total protein, albumin	Less hepatocyte vacuolation and cellular regeneration	[23]

4. *Argemone Mexicana* L.	Aerial parts	Methanol, aqueous (100, 200, and 400 mg/kg)	CCl_4_-induced	SGOT, SGPT, ALP	100 mg/kg methanolic extract more effective in healing liver parenchyma than other doses; 400 mg/kg aqueous extract showed a good result than the corresponding dose of methanol extract	[24]

5*. Balanites aegyptiaca* L.	Bark	Water, methanol (250 and 500 mg/kg)	CCl_4_-induced	AST, ALT, ALP, bilirubin	Both extracts decrease centrilobular degenerative changes	[25]

6. *Cannabis sativa* L.	Seeds	Petroleum ether (1 and 0.5 ml/kg)	CCl_4_-induced	AST, ALT, ALP, bilirubin	1 ml/kg: showed scattered areas of necrosis of the hepatocytes	[26]
					0.5 ml/kg: showed slight areas of necrosis and slight fatty change	

7. *Capparis decidua* (Forsk.)	Stems	Aqueous, methanol (200 and 400 mg/kg)	CCl_4_-induced	AST, ALT, ALP, bilirubin	Aqueous and methanolic extracts masked the liver fatty changes	[7, 27]
	Branches	Methanol (12.5, 25, and 50 mg/kg)	CCl_4_-induced, paracetamol-induced	AST, ALT, ALP, bilirubin, total protein, albumin	—	[28]
	Roots	Ethanol (200 mg/kg)	CCl_4_-induced	AST, ALT, ALP, bilirubin, total protein, albumin	Less hepatocyte vacuolation and cellular regeneration	[23]

8. *Combretum hartmannianum* (Schweinf)	Leaves	Methanol (12.5, 25, and 50 mg/kg)	CCl_4_-induced, paracetamol-induced.	AST, ALT, ALP, bilirubin, total protein, albumin	—	[28]

9. *Dobera glabra* (Forsk.)	Leaves	Aqueous methanol (200 and 400 mg/kg)	CCl_4_-induced	AST, ALT, ALP, bilirubin	Diffuse centrilobuar necrotic lesions in liver cells	[29]

10. *Khaya senegalensis* (Desr.)	Bark	Aqueous (250 and 500 mg/kg)	CCl_4_-induced	AST, ALT, ALP, bilirubin, total protein, albumin	250 mg/kg: showed moderate necrosis of the hepatocytes and vacuolations	[30, 31]
					500 mg/kg: almost mild fatty changes in livers	
	Bark	Ethanol (200 mg/kg)	CCl_4_-induced	AST, ALT, ALP, bilirubin, total protein, albumin	Mild histopathological changes in the liver	[23]
	Bark	Methanol (12.5, 25, and 50 mg/kg).	CCl_4_-induced	AST, ALT	—	[32]
	Bark	Methanol (50 mg/kg)	paracetamol-induced	AST, ALT, ALP, bilirubin, total protein, albumin	Section of methanolic extract-treated rats showing few vacuolation, reduced sinusoidal dilatation, and no congestion	[33]

11. *Kigelia africana* Lam.	Seeds	Aqueous (400 mg/kg), methanol (100, 200, and 400 mg/kg)	CCl_4_-induced	AST, ALT, ALP	Cytoplasmic fatty vacuolation, haemorrhages, and necrosis of the centrilobular hepatocytes	[34]

12. *Lawsonia inermis* L.	Leaves	Methanol (100 and 200 mg/kg)	CCl_4_-induced	AST, ALT, ALP, bilirubin, total protein	Showed minimal necrosis and regeneration of hepatocytes compared with CCl_4_	[35]

13. *Lepidium sativum* L.	Seeds	Methanol (200 and 400 mg/kg)	CCl_4_-induced	AST, ALT, ALP, bilirubin.	Mild-to-moderate changes in hepatocytes in both doses	[36]

14. *Moringa oleifera* Lam.	Leaves	Aqueous (200 and 400 mg/kg)	CCl_4_-induced	AST, ALT, ALP, bilirubin	Slight changes in liver cells	[37]

15. *Nigella sativa* L.	Seeds	Methanol (250 and 500 mg/kg)	CCl_4_-induced	AST, ALT, ALP	Less centrilobular vacuolation and inflammatory cell infiltration.	[38]
	Seeds	Aqueous (250 and 500 mg/kg)	CCl_4_-induced	AST, ALT, ALP, bilirubin, total protein	Less hepatocyte changes	[30]
	Seeds	Methanol (25, 50, 75, and 100 mg/mL) *in vitro*	Paracetamol-induced	Blood ions, ALT, AST, ALP, antioxidant enzymes, e.g., SOD, GSH, and MDA	Improvement of lesions observed in CCl_4_ such as necrosis, loss of hepatocytes architecture, and cellular infiltration	[39]
		Methanol (100, 300, and 900 mg/kg) *in vivo*				

16. *Occimum basilicum* L.	Whole plant	Ethanol (200 mg/kg)	CCl_4_-induced	AST, ALT, ALP, bilirubin, total protein, albumin	Less vacuolated hepatocytes and cellular regeneration	[40]

17. *Phoenix dactylifera* Linn.	Pollen grain powder	Water (250 and 500 mg/kg)	CCl_4_-induced	AST, ALT, ALP	Mild changes in hepatocytes	[41]

18. *Raphanus sativus* L.	Seeds	Methanol, water (200 and 400 mg/kg)	CCl_4_-induced	AST, ALT, ALP, bilirubin	Mild-to-moderate changes in hepatocytes in both doses	[42]

19. *Solanum nigrum* L.	Whole plant	Water, methanol (250 and 500 mg/kg)	CCl_4_-induced	AST, ALT, ALP, bilirubin	Water: small vacuoles were seen in centrilobular hepatocytes. In methanol, slight changes in the fat content of hepatocytes were seen.	[43]

20. *Sterculia Setigera* Del.	Bark	Ethanol, ethyl acetate (200 and 400 mg/kg)	CCl_4_-induced	AST, ALT, ALP, bilirubin, total protein, albumin	Showed no stenosis (fatty changes in hepatocytes) and perivenular fibrosis compared with the CCl_4_ group.	[44]

21. *Tamarindus indica* L.	Fruit's pulp	Ethanol (150 mg/kg)	CCl_4_-induced	AST, ALT, ALP, bilirubin	Showed slight fatty changes	[45]

**Table 2:** Families, local names, distribution, traditional uses, and chemical constituents of hepatoprotective plants.

Plants	Family	Local name	Distribution in Sudan	Traditional uses	Chemical constituents	References
1. *Acacia mellifera* (Vahl)	Fabaceae	Keka/kitir	Northern and Central Sudan	Bowel problems, stomach trouble, cold, treatment for malaria and inflammation	Alkaloids, flavonoids, tannins, sterols, and saponins	[2, 21, 46]

2. *Adansonia digitata* L.	Malvaceae	Baobab tree, tabaldi, fruit (gonglize)	Widespread and throughout Northern and Central Sudan, Kordofan, Darfur	Immunostimulant, anti-inflammatory, analgesic, pesticide, antipyretic, febrifuge, and astringent in the treatment of diarrhea	Terpenoids, flavonoids, steroids, vitamins, amino acids, carbohydrates, and lipids	[15, 16, 22, 47]

3. *Argemone Mexicana* L.	Papaveraceae	Khash khash	Northern Sudan	Analgesic, antispasmodic, possibly hallucinogenic, and sedative, tumors, warts, skin diseases, inflammations, rheumatism, jaundice, leprosy, piles, warm infestations, and dysentery	Alkaloids as berberine, protopine, sarguinarine, optisine, and chelerytherine; the seed oil contains myristic, palmitic, oleic, and linoleic acids; flavonoids, glycosides, sterols, and phenolic compounds (tannins)	[24, 48, 49]

4. *Anogeissus leiocarpus* (DC. Wall)	Combretaceae	Sahib	Kassala, Kordofan, Darfur	Parasitic diseases and dysentery	Rich in derivatives of ellagic acid, polyalcohol sorbitol, terpenoids (*α*-amyrin, *β*-amyrin and *β*-sitosterol), and traces of alkaloids	[15, 23, 50, 51]

5. *Balanites aegyptiaca* L.	Balanitaceae	Hegleig tree (Ar), laloub fruit	Widespread. and throughout Northern and Central Sudan	Jaundice, liver disorders, and spleen problems; the leaves and branches are used as a fumigant for rheumatism; fruits are used aginst constipation and as an antidiabetic	The root contains rotenone and yamogenin; bark contains steroidal sapogenins	[15, 16, 25, 52, 53]

6. *Cannabis sativa* L.	Cannabaceae	Bango, hashish.	Cultivated in various areas in Sudan	Spasmolytic, hypnotic, analgesic to treat rabies, cholera, rheumatism, epilepsy, and tetanus; anti-inflammatory	Cannabinoids (tetrahydrocannabinol (THC)), cannabidiol (CBD) and cannabinol (CBN) are the most prevalent natural cannabinoids)	[26]

7. *Capparis decidua (Forsk.)*	Capparaceae	Tundub	Northern and central Sudan; Western and eastern Sudan	Jaundice, antirheumatic diuretics, antigout, anti-inflammatory, astringent, stomachic, laxative, antidote, and used for skin diseases	Alkaloids, saponins, flavonoids, tannins, sterols, cyanogenic glycosides and cumarins	[7, 15, 16, 27]

8. *Combretum hartmannianum* (Schweinf)	Combretaceae	Habiel, Habeel Al Gabal	El-Jebelein. White Nile, Kordofan, Darfur, Blue Nile	Boiled leaves used to cure ascites; bark and leaves extract are used for jaundice	Alkaloids, flavonoids, tannins hydrocyanic acid, and phenanthrene derivatives	[1, 2, 15, 28]

9. *Dobera glabra* (Forsk.)	Salvadoraceae	Al meikah	Widespread. Northern Sudan, Kordofan, Darfur, Khartoum	Ophthalmic problems	Alkaloids, saponins, flavonoids, tannins, sterols, cyanogenic glycosides and cumarins	[2, 15, 29]

10. *Khaya senegalensis* (Desr.)	Meliaceae	Mahogany tree	Darfur	Dysentery, diarrhea, and wound infections, fever, and remedy to treat syphilis; the bark is used in jaundice, scorpion bites, allergies	Saponins, tannins, alkaloids, glycosides, steroids, terpenoids flavonoids, and phenolic compounds	[23, 54]

11. *Kigelia africana* Lam.	Bignoniaceous	Umm Shutour	Central Sudan, Kassala, Kordofan, Blue Nile	Bark is used for ulcers treatment or for treatment of pneumonia and malaria	Verminosides and iridoids and series of polyphenols such as verbascoside	[2, 15, 34]

12. *Lawsonia inermis* L.	Lythraceae	Henna	Widespread especially in Northern and Central Sudan	Astringent, hypotensive, sedative, and against headache, jaundice, leprosy, and skin diseases	Naphthoquinone, phenolic derivatives, coumarins, xanthones, tannins, flavonoids, aliphatic components, triterpenes, sterols glucose, gallic acid, amino acids, mannitol, trace elements, and minerals	[2, 15, 35]

13. *Lepidium sativum* L.	Brassicaceae	Pepper cress or ELRshad	Central Sudan	Gastrointestinal disorders, arthritis, and inflammatory disorders dysentery and diarrhea	Triterpenes, alkaloids, flavanoids, tannins, coumarins, and saponins	[2, 36]

14. *Moringa oleifera* Lam.	Moringiaceae	Al-rawag	Widespread, Northern and Central Sudan	Liver disease, lipid disorders, arthritis, and inflammatory disorders; seeds used to clean water for drinking	Alkaloids, saponins, flavonoids, tannins, sterols, glycosides, and cumarins	[2, 15, 37]

15. *Nigella sativa* L.	Ranunculaceae	Black seed, black cumin, or habat elbarka	Northern Sudan, Darfur (Melit and Jebel Marra)	Liver tonics, digestive, anti-inflammatory, immunostimulant, and remedy for jaundice, antidiabetics	Thymoquinone, thymohydro quinine, dithymoquinone, p-cymene, carvacrol, and 4-terpineol	[38]

16. *Occimum basilicum* L.	Lamiaceae	Alryhan	Widespread throughout Northern and Central Sudan	Jaundice, stomach complaints, fever, cough, and gout, diuretic, aphrodisiac, and antidysenteric actions; the seeds are used as demulcents	Glycoside, gums, mucilage, proteins, amino acids, tannins, phenolic compound, triterpenoids steroids, sterols, saponins, flavones, and flavonoids	[16, 40, 55]
					Linalol methylchavikol, methylcinnamat, and linolen, essential oil, rosmarinic acid, citral, eugenol, and geraniol	

17. *Phoenix dactylifera* Linn.	Palmae	Date, date palm	Northern Sudan, most parts of the Sudan	Sexual incapacity and weakness	Carbohydrates, alkaloids, steroids, flavonoids, vitamins tannins, and phenolic acids	[15, 41, 56]

18. *Raphanus sativus* L.	Cruciferae	Alfgel.	Widespread	Hepatoprotective, bacterial and viral infections, inflammation	Flavonoids, saponins, cumarins, and alkaloids	[42]

19. *Solanum nigrum* L.	Solanaceae	Enab eldib	Widespread	Inflammatory disorders, rheumatism and swellen joints, hepatomegaly, splenomegaly, edema, gonorrhea	Gallic acid, catechin, protocatechuic acid, caffeic acid, epicatechin, rutin, and naringenin	[43, 57]
			Poisonous plants in the Sudan			

20. *Sterculia setigera* Del.	Sterculiaceae	Terter	Blue Nile, Kassala, Kordofan, Nuba mountains, Darfur and red Sea hill regions	Jaundice and bilharzia	Saponins, steroidal, sterols, and flavonoids	[15, 44, 58]
					Tannins, saponins, cardiac glycosides, and anthraquinone	

21. *Tamarindus indica* L.	Caesalpinaceae	Aradaib.	Central Sudan	To treat fever, postpartum remedy, and measles	Tannins and alkaloids and low concentration of flavonoids and saponins	[15, 45, 59, 60]
